# To contact or not: an investigation into the polymeric surface interactions with human insulin and their therapeutic implications

**DOI:** 10.1039/d5ra08501b

**Published:** 2026-02-12

**Authors:** Megren H. A. Fagihi, Chanaka Premathilaka, Laura Zopf, Tiina OʼNeill, Massimiliano Garré, Sourav Bhattacharjee

**Affiliations:** a School of Medicine, University College Dublin Belfield, Dublin 4 Ireland; b Conway Institute of Biomolecular and Biomedical Research, University College Dublin Belfield, Dublin 4 Ireland sourav.bhattacharjee@ucd.ie +353 1 716 6271; c Clinical Laboratory Sciences Department, College of Applied Medical Sciences, Najran University Najran 55461 Kingdom of Saudi Arabia; d Institute of Veterinary Medicine and Animal Sciences, Estonian University of Life Sciences Tartu 51006 Estonia; e School of Veterinary Medicine, University College Dublin Belfield, Dublin 4 Ireland; f Super-Resolution Imaging Consortium, Royal College of Surgeons in Ireland University of Medicine and Health Sciences Dublin D02 YN77 Ireland; g UCD One Health Centre, University College Dublin Belfield, Dublin 4 Ireland; h UCD Earth Institute, University College Dublin Belfield, Dublin 4 Ireland; i Institut für Funktionelle Anatomie, Charité – Universitätsmedizin Berlin, Campus Charité Mitte Philippstraße 11 10115 Berlin Germany

## Abstract

Insulin, a therapeutic peptide used to treat Type I diabetes, interacts with polymers commonly found in healthcare settings *via* various hydrophobic interactions, which can trigger insulin agglomeration, thereby reducing its bioavailability and therapeutic efficacy. In this study, a fluorescein isothiocyanate (FITC)-labeled human insulin (*λ*_ex_ = 490 nm; *λ*_em_ = 498–530 nm) suspension (0.125 mg mL^−1^, 0.25 mg mL^−1^, and 0.5 mg mL^−1^) prepared at pH 3 was interacted with fluorescent amine- (*λ*_ex_ = 560 nm; *λ*_em_ = 570–650 nm) and acid-terminated (*λ*_ex_ = 625 nm; *λ*_em_ = 640–720 nm) polystyrene particles (1 µm) at 37 °C for *t* = 2 h, 4 h, 24 h, 48 h, and 72 h. The variable fluorescence lifetime of FITC, driven by pH fluctuations, was used as a *molecular pH meter* to map pH alterations within insulin agglomerates. Larger agglomerates, with higher lifetime variations, were noticed for the amine-terminated particles, especially at longer timepoints, whereas such fluctuations were relatively subtle in the acid-terminated ones. Regions with lifetime variation spread beyond the adsorbed insulin layer on particles and merged with the peripheral zones of lower lifetimes inside the agglomerates. The results suggest that polymeric surfaces alter insulin's biochemical properties, with probable implications of reduced bioactivity, poor glycemic control, and (potential) additional side effects.

## Abstract 2

Insulin, a therapeutic peptide for Type I diabetes, can interact with polymers commonly found in healthcare settings *via* hydrophobic interactions, potentially triggering agglomeration while reducing its bioavailability and therapeutic efficacy.

## Graphical abstract

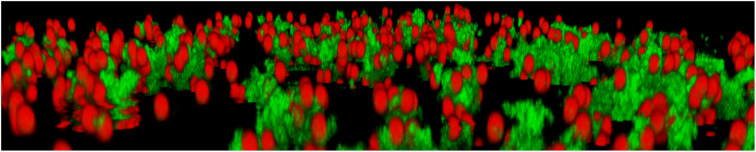

## 1. Introduction

**Fig. 1:**
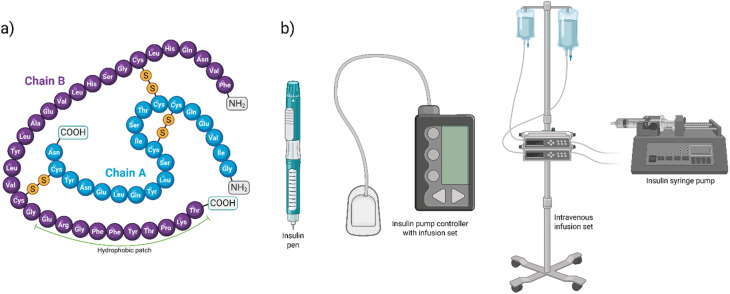
(a) Scheme showing a human insulin molecule with its A and B chains. The A and B chains carry 21 and 30 amino acids, respectively, and are linked by two interchain disulfide linkages (A7–B7 and A20–B19). The A chain also has an intrachain disulfide bridge (A6–A11). The C-terminus of the B-chain exhibits a hydrophobic patch due to ten (hydrophobic) amino acid residues (Glu–Arg–Gly–Phe–Phe–Tyr–Thr–Pro–Lys–Thr) arranged in tandem, and is marked within the figure. (b) Scheme showing the various polymeric surfaces that the human insulin comes into contact under therapeutic setups.

As diabetes cases reach unprecedented global levels—now affecting more than 830 million people of all ages^1,2^—the importance of insulin as a first-line therapy for insulin-dependent (Type I) diabetes has soared markedly in recent decades.^3^ Insulin, a peptide hormone with a molecular weight of 5808 Da, is synthesized, processed, and stored as a (physiologically inactive) hexamer in the β-cells of the pancreatic islets of Langerhans.^4^ It is released into the bloodstream in response to elevated blood sugar levels, such as after a meal. Upon release, the hexameric insulin rapidly unfolds into oligomers in the blood before finally yielding the physiologically active monomer, which lowers the blood sugar.^5^ Structurally, insulin comprises an A chain (21 amino acids) and a B chain (30 amino acids), connected by two interchain disulfide bonds, while the A chain contains an intrachain disulfide bridge (Fig. 1a).

Interestingly, the C-terminus of insulin's B-chain has ten hydrophobic amino acid residues arranged in tandem to form a hydrophobic patch (Fig. 1a), and renders it prone toward interactions with a range of molecules, chiefly the polymeric ones, which are now ubiquitous in healthcare set up—from infusion bottles to syringes and tubings, or even the latest insulin delivery pumps (Fig. 1b).^4,5^ There are sporadic reports on interactions between insulin and hydrophobic surfaces with a propensity toward insulin agglomeration and (amyloid) fibrillation.^6–8^ However, whether such (surface) interactions actually interfere with the physicochemical attributes of insulin molecules remains underexplored, despite its relevance in therapeutics, as any molecular alteration of insulin risks affecting its behavior from a pharmacological context, including receptor binding—crucial for its bioactivity^9–11^—in a deleterious manner. From a therapeutic perspective, it is important, given that a firm grip over insulin dosing is of paramount importance in diabetes management, while it is equally important to have an accurate estimation of the amount of insulin that is bioactive or bioavailable to the patients upon administration.

With an interest in insulin therapeutics, our lab has published multiple reports on the molecular behavior of insulin when subjected to a diverse range of physicochemical alterations, including fluctuations in pH and temperature.^4,5^ We have established and optimized a range of biophotonic platforms, including confocal laser scanning microscopy (CLSM) and fluorescence lifetime imaging microscopy (FLIM), to investigate fluorescein isothiocyanate (FITC)-labeled insulin, including its agglomerates. The fluorescence lifetime (*τ*) of unbound FITC in water is 4 ns, although it can be ∼3.7 ns in conjugated states.^12^ Interestingly, this lifetime of FITC varies depending on its microenvironment pH: a lower lifetime is observed in acidic pH and *vice versa*. Thus, the fluorescence lifetime of FITC can be used as a *molecular pH meter* to estimate the local pH in an insulin suspension or to map segments with diverse pH within insulin agglomerates.^13^ The high sensitivity of such biophotonic platforms provides molecular-level understanding of insulin's behavior in biochemically complex and fluctuating systems, which is often the case inside the human body.

On the other hand, polystyrene is a widely used synthetic hydrophobic polymer with a diverse range of applications in daily life. Regarding insulin delivery, polystyrene has multiple uses, including the manufacture of various equipment (*e.g.*, plastic bottles, tubing) and medical devices.^14^ It is popular in pharmacy labs, with its use now prevalent in making test tubes, Petri dishes, diagnostic gadgets, and packaging materials.^15,16^ Polystyrene's affordability, availability in various forms, hardness, and ease of sterilization have made it a polymer of choice for a wide range of applications in pharmaceutics, including insulin formulation preparation and delivery. Thus, it is a model polymer for investigating interactions between insulin and therapeutically relevant polymers. Interestingly, polystyrene is also available in both micro-^17^ and nanoparticle^18^ forms. These particulate preparations are inexpensive, stable, monodisperse, spherical, and available in bulk quantities. They often carry diverse surface functionalizations and are frequently tagged with multiple fluorophores, enabling a wide range of biophotonic investigations. Such a highly useful cohort of fluorescent polystyrene particulates has naturally established its niche in biomedical investigations, including advanced light microscopy.^19,20^

Here, we leveraged the availability of well-characterized fluorescent polystyrene microparticles to provide functionalized surfaces for an insulin interaction study. Additionally, different surface functionalization of these particles imparted various (pH-dependent) surface charges (cationic or anionic) in aqueous dispersions. Finally, their precise spherical geometry enabled the calculation of particle surface areas and helped in sustaining uniformity across different experimental repetitions. Our driving hypothesis was that polystyrene particles with comparable composition and geometry but different surface functionalizations would exert varying influences on FITC-insulin photochemistry. The fluorescence of these particles, with their emission not overlapping with that of FITC-insulin, provided a unique opportunity to investigate how the particulate surfaces interacted and influenced insulin's molecular integrity, which is relevant to its physiological activity. The photostability of both FITC-insulin and the particulate dyes, in conjunction with the high sensitivity of CLSM and FLIM, further strengthened the rationale behind the study.

The aims and objectives of this study were: (i) to procure commercially available fluorescent amine- and acid-terminated polystyrene microparticles (1 µm); (ii) to characterize these particles with the help of scanning electron microscopy (SEM), nanoparticle tracking analysis (NTA), and zeta potential (ZP); (iii) to expose FITC-insulin suspensions of various concentrations to these microparticles (100 µg mL^−1^) for different timepoints; (iv) to visualize the FITC-insulin agglomerates—with or without entrapped polystyrene particles—with the help of CLSM; (v) to conduct FLIM and comprehend how the surface exposure altered the biochemistry of insulin by creating segments of various lifetimes (indicative of pH-fluctuation) inside the FITC-insulin agglomerates; (vi) to compare the amine- and acid-terminated particles, and figure out if diverse surface functionalizations impacted such surface–insulin interactions.

The obtained data highlighted the importance of surface functionalization in determining how surfaces interact with FITC-insulin, while delving deeper into the crucial molecular mechanisms that drive such interactions. Furthermore, the cumulative data provided insights into how such surface interactions could influence insulin's physiological activity, while also identifying ways to limit them.

## 2. Materials and methods

### 2.1. Chemicals and reagents

FITC-labeled recombinant human insulin was commercially procured from Sigma Aldrich (product number: I3661; degree of substitution: 1 mole mole^−1^). The lyophilized powder was preserved in the dark (−20 °C), and suspended in a pH 3 solution (0.125 mg mL^−1^, 0.25 mg mL^−1^, and 0.5 mg mL^−1^) at 37 °C.

### 2.2. Polystyrene particles

Fluorescent amine- (Sigma Aldrich, Product Number: L2778, 2.5% solid) and acid-terminated (ThermoFisher Scientific, Part Number: F8816, 2% solid) polystyrene particles, both 1 µm in size, were obtained commercially and were used as received.

### 2.3. Particle characterization

The particles were imaged using a Zeiss Sigma 300 SEM microscope after sputter-coating the samples with platinum. The particle (hydrodynamic) radius was measured by nanoparticle tracking analysis (NTA) using a ZetaView PMX 110 V3.0 instrument (Particle Metrix GmbH, Germany). The instrument was calibrated with a known concentration of 100 nm polystyrene nanoparticles (Applied Microspheres B.V., Netherlands). The polystyrene microparticles were diluted in a pH 3 solution for the NTA measurements. The size distribution was determined from three cycles of measurements (11 frames per cycle, sensitivity = 85, and shutter value = 100). Similarly, the ZP was measured thrice (*n* = 3) at 25 °C (30 frames per s, sensitivity = 85, and shutter value = 70), while ZetaView software was used to collect and analyze the data.

### 2.4. Experimental design

Aliquots of 1 mL from FITC-insulin suspensions (0.125 mg mL^−1^, 0.25 mg mL^−1^, and 0.5 mg mL^−1^) at pH 3 were taken in Eppendorf tubes and mixed with amine- or acid-terminated polystyrene particles (final particle concentration = 100 µg mL^−1^), before being shaken at 300 rpm at 37 °C in a Thermomixer instrument for *t* = 2 h, 4 h, 24 h, 48 h, and 72 h. All experiments were conducted in triplicates (*n* = 3).

### 2.5. Microscopy

Aliquots of 300 µL volumes from the FITC-insulin-particle mixes were deposited in an eight-chamber microslide with glass coverslip bottoms (Cat. No: 80821, ibidi GmbH, Germany) for CLSM and FLIM investigations at room temperature (21 °C) in a Leica Stellaris 8 Falcon system fitted with LAS X software (version 4.4.0.24861). The acquisition was performed with a Leica HC PL APO CS2 100×/1.40 oil-immersion objective and a Leica HyD S detector. The different wavelengths used for the acquisition were: FITC-insulin (*λ*_ex_ = 490 nm; *λ*_em_ = 498–530 nm), amine-terminated particles (*λ*_ex_ = 560 nm; *λ*_em_ = 570–650 nm), and acid-terminated particles (*λ*_ex_ = 625 nm; *λ*_em_ = 640–720 nm). The pixel size (<100 nm) was determined based on the Nyquist sampling theorem to achieve the highest image resolution. The *z*-stack acquisitions, FLIM analyses, and image processing were performed using the Leica LAS X software. 3D reconstructions from *z*-stacks in both the CLSM and FLIM acquisitions were performed.

## 3. Results

### 3.1. Scanning electron microscopy

**Fig. 2:**
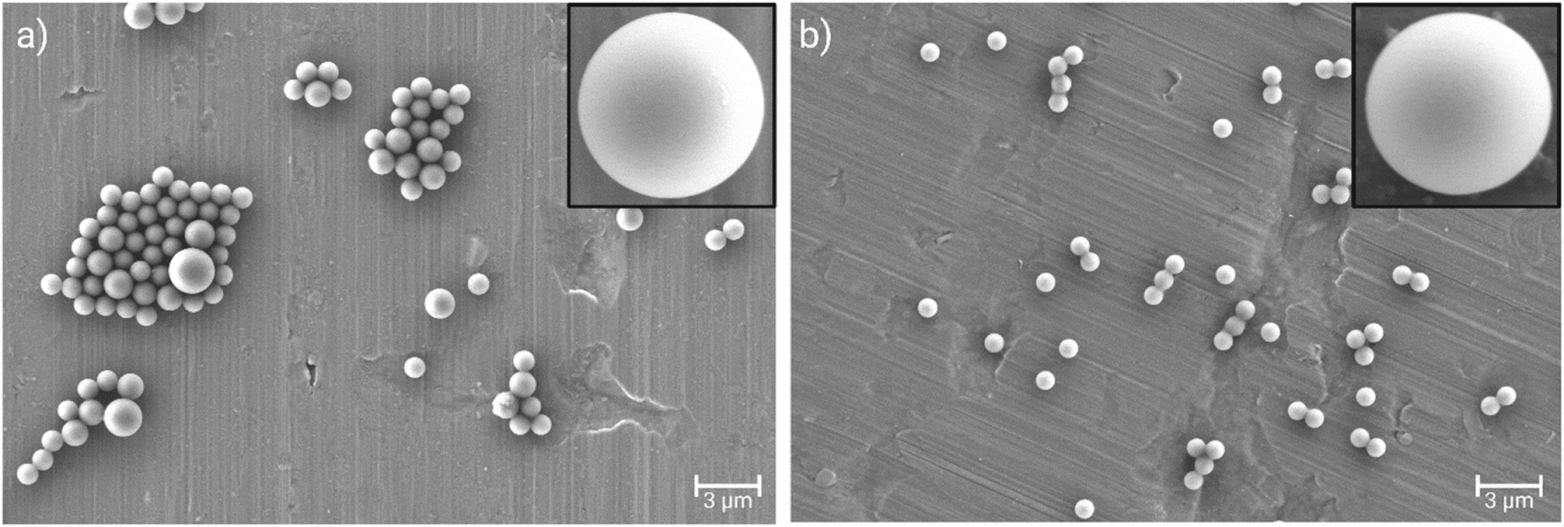
The SEM images (×45 000) of the (a) amine- and (b) acid-terminated polystyrene particles. Inset images provide a zoomed view (2 500 00×) of an individual particle. A 3 µm scale bar is shown.

The SEM confirmed the presence of monodisperse spherical particles (both amine- and acid-terminated) of ∼1 µm diameters (Fig. 2).

### 3.2. NTA and ZP

**Fig. 3:**
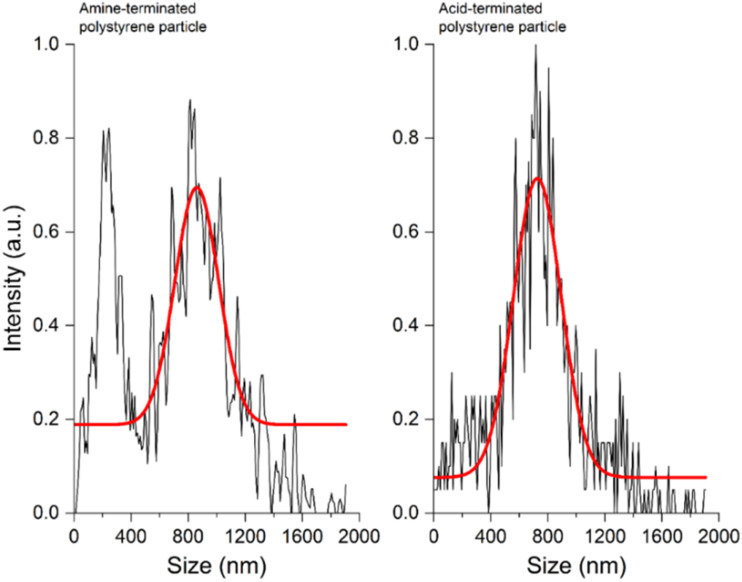
The NTA data on amine- (left) and acid-terminated (right) polystyrene particles showing their hydrodynamic diameters at pH 3. The data were normalized (0–1), followed by smoothing (Savitzky–Golay method), and fitted in a non-linear curve fitting tool with a Gaussian fit (red trace).

The hydrodynamic diameters of these particles, when checked by NTA (Fig. 3), corroborated the SEM data. The mean (*n* = 3) ZP of the amine-terminated particles in water and at pH 3 were 6.47 mV and 9.89 mV, respectively. Similarly, for the acid-terminated particles, the values were −10.89 mV (water) and 3.22 mV (pH 3).

### 3.3. Particulate surface area calculation

With a diameter of 1 µm, the surface area and volume of each particle were ∼3.14 µm^2^ and ∼0.524 µm^3^, respectively. Assuming the density of polystyrene to be 1 g.cm^−3^, the mass of each particle was ∼5.24 × 10^−7^ µg. Hence, there were 100 µg ÷ (5.24 × 10^−7^ µg) = 1.9 × 10^8^ particles per mL volume of particle–insulin mix, that is, in each Eppendorf. Thus, the total surface area available to the FITC-insulin in each Eppendorf containing 1 mL aliquot was 1.9 × 10^8^ × 3.14 µm^2^ = 6 × 10^8^ µm^2^ = 6 × 10^−4^ m^2^.

### 3.4. Confocal laser scanning microscopy

**Fig. 4:**
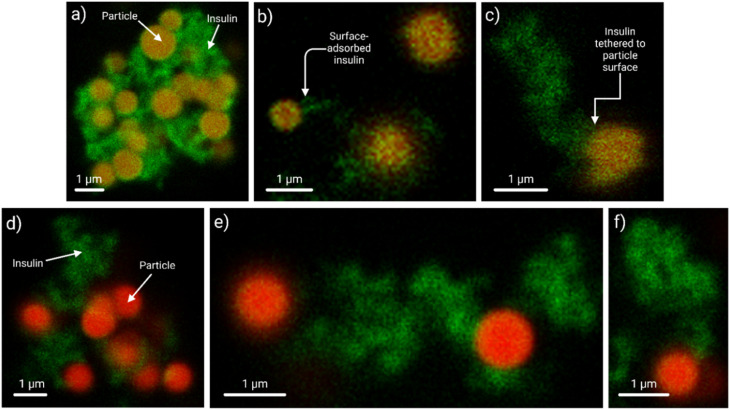
CLSM data showing how the spherical (a–c) amine- (*λ*_ex_ = 560 nm; *λ*_em_ = 570–650 nm), and (d–f) acid-terminated (*λ*_ex_ = 625 nm; *λ*_em_ = 640–720 nm) polystyrene microparticles interacted with the FITC-insulin (*λ*_ex_ = 490 nm; *λ*_em_ = 498–530 nm). Panels (a) and (d) show larger FITC-insulin agglomerates (0.5 mg mL^−1^, *t* = 72 h) with multiple trapped amine-or acid-terminated particles. The amine-terminated particles formed larger agglomerates over time. Panels (b) and (e) show the interactions between FITC-insulin (0.25 mg mL^−1^, *t* = 24 h) and a few amine- or acid-terminated particles, while panels (c) and (f) show the FITC-insulin (0.125 mg mL^−1^, *t* = 48 h) interaction with individual amine- or acid-terminated particles. A scale bar of 1 µm is shown in each panel.

The CLSM data demonstrated the particle–insulin interactions (Fig. 4, Video Files S1 (3D rendition of the interactions between a FITC-insulin agglomerate (0.25 mg mL^−1^) and amine-terminated polystyrene particles (*t* = 48 h), and S2 (3D rendition of FITC-insulin agglomerates (0.25 mg mL^−1^) with entrapped acid-terminated polystyrene particles (*t* = 48 h)). While multiple particles were trapped inside larger agglomerates (Fig. 4a and d), surface adsorption of FITC-insulin on the particles was identified (Fig. 4b and c). The amine-terminated particles formed larger insulin agglomerates than the acid-terminated ones, with an overall concentration- and time-dependent effect: at longer time points (*t* = 48 h, 72 h) and higher concentrations (*e.g.*, 0.5 mg mL^−1^), larger agglomerates formed. FITC-insulin trails tethered to the particles were also noted (Fig. 4c and f).

### 3.5. Fluorescence lifetime imaging microscopy (FLIM)

#### 3.5.1. Amine-terminated particles

**Fig. 5:**
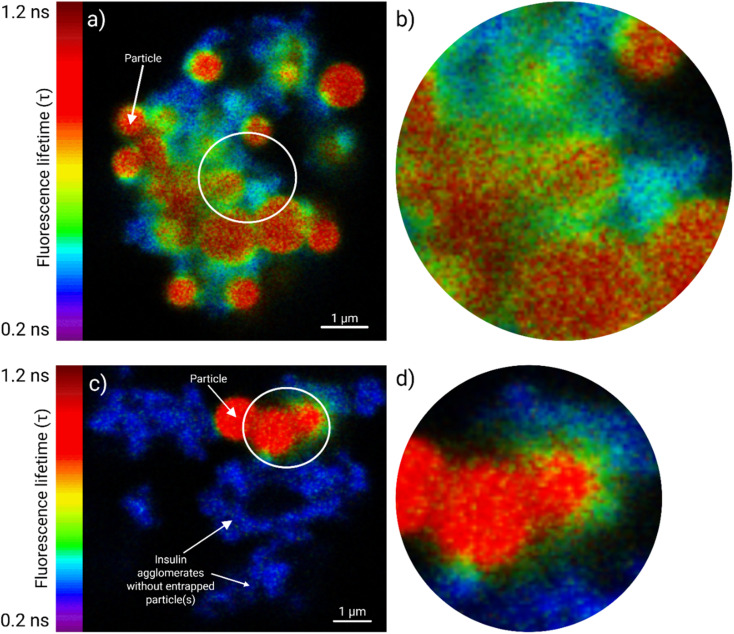
The FLIM study elucidated the interactions between FITC-insulin and fluorescent amine-terminated polystyrene particles (1 µm), and the data are presented as a fluorescence lifetime heatmap spanning 0.2–1.2 ns. Panel (a) shows the fluorescence lifetime (*τ*) distributions inside a FITC-insulin agglomerate (0.5 mg mL^−1^, *t* = 72 h) with multiple trapped (spherical) particles. The FITC-insulin layers adhered to particle surfaces exhibited longer lifetimes (∼1 ns), which gradually merged with the peripheral domains of lower lifetimes through transitional intermediary lifetimes. A region of interest (white circle) was further zoomed in panel (b) for granular viewing of the various lifetime regions. Panel (c) shows another FITC-insulin agglomerate (0.125 mg mL^−1^, *t* = 48 h) with fewer trapped particles, while a similar region of interest, enclosed in a white circle, is magnified in panel (d). Agglomerates without entrapped particles are also shown, which did not elicit a lifetime variation, and confirmed that such lifetime fluctuations were exclusively due to (amine-terminated) particle contact. A scale bar of 1 µm is provided for both panels (a) and (c).

The amine-terminated polystyrene particles led to greater fluorescence lifetime fluctuations within the FITC-insulin agglomerates (Fig. 5). The FITC-insulin in close contact with the aminated particles due to surface adsorption showed higher fluorescence lifetimes (*τ*) of ∼1 ns (Fig. 5a and c), while the higher lifetime regions gradually merged with areas of shorter lifetimes at a distance from particles (Fig. 5b and d). The FLIM data enabled visualization of these diverse τ-regions within insulin agglomerates containing multiple entrapped amine-terminated particles and mapping of their spatial distribution. Only FITC-insulin agglomerates (Fig. 5c) without trapped particles did not show lifetime fluctuation (*τ* ∼ 0.3 ns).

#### 3.5.2. Acid-terminated particles

**Fig. 6:**
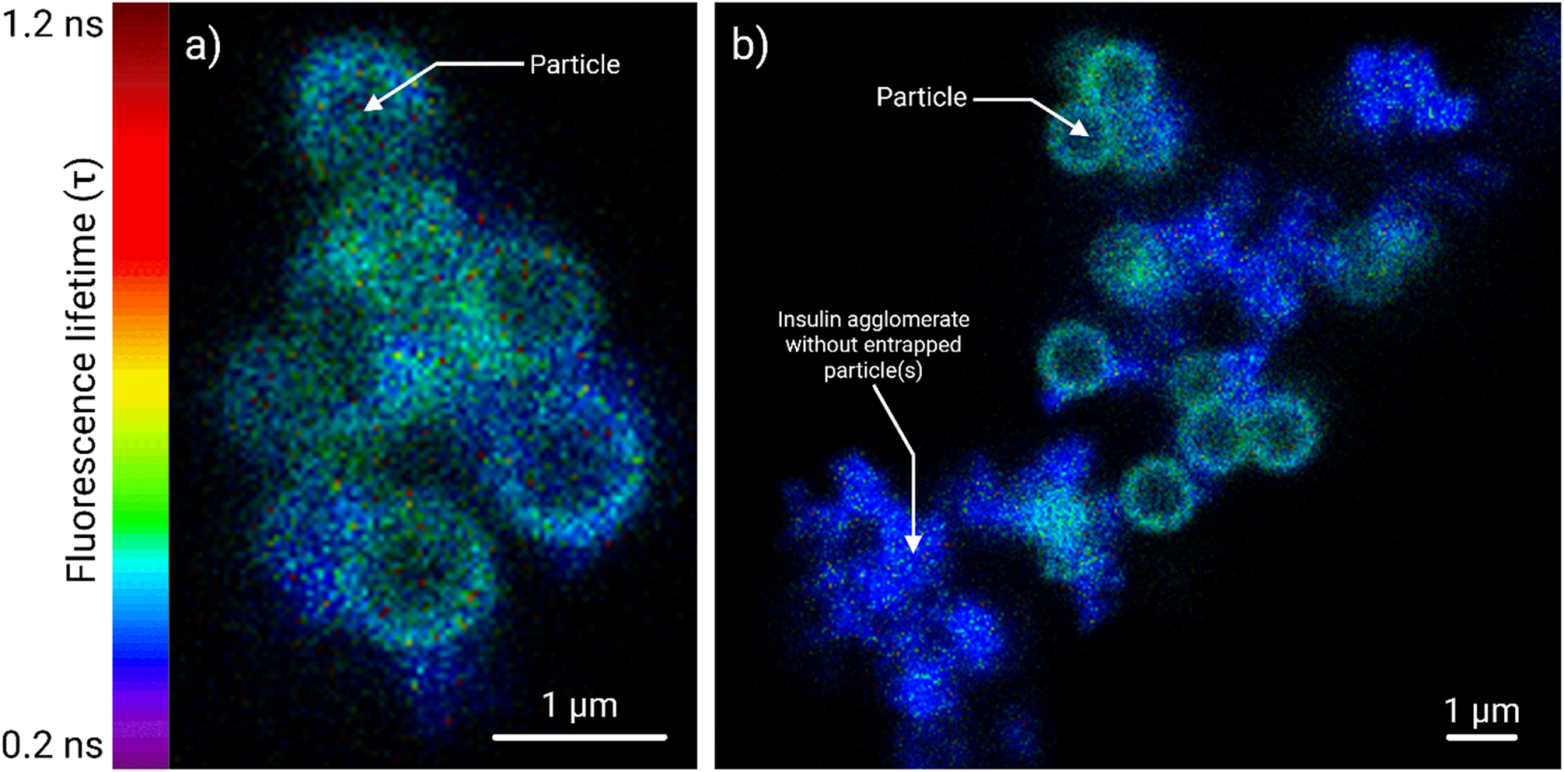
Compared to their amine-terminated counterparts, the fluorescence lifetime variation inflicted by the acid-terminated particles (1 µm) in FITC-insulin agglomerates was rather subtle. The lifetime data is presented as a heatmap, scaled from 0.2 to 1.2 ns. Panel (a) shows the fluorescence lifetime (*τ*) distributions inside a FITC-insulin agglomerate (0.5 mg mL^−1^, *t* = 72 h) with multiple trapped acid-terminated particles. Contact with these particles induced some variation in FITC's fluorescence lifetime, especially at particle surfaces. However, unlike the amine-terminated particles, no transitional regions of fluctuating and mixed lifetimes were observed. Panel (b) shows another field of FITC-insulin agglomerates (0.125 mg mL^−1^, *t* = 48 h) with fewer trapped (acid-terminated) particles. FITC-insulin agglomerates without particles served as a control (*τ* ∼ 0.3 ns). A scale bar of 1 µm is provided for the panels (a) and (b).

On the contrary, the acid-terminated particles did not cause any significant fluorescence lifetime fluctuation within the insulin agglomerates (Fig. 6). When multiple acid-terminated particles were entrapped in larger agglomerates (Fig. 6a), the overall FITC lifetime varied between 0.4 and 0.6 ns. The adsorbed FITC-insulin layer on the particles showed slightly higher lifetimes (*τ* ∼ 0.45 ns), while the variation otherwise within the agglomerates was low (<0.2 ns). Additionally, the fluorescence lifetime variation was more uniform compared to the amine-terminated particles (Fig. 6b).

## 4. Discussion

The fluorescent polystyrene particles served as an adequate biophotonic tool for probing FITC-insulin interactions, with precise mapping of the different lifetime regions within insulin agglomerates. Such mapping enabled visualization of fluorescence lifetime fluctuations, especially when the FITC-insulin interacted with the amine-terminated particles. The stability of these particles enabled detailed analysis of the data and identification of how the particle surfaces interacted with FITC-insulin. The known particle diameter (1 µm) served as a convenient internal scale during CLSM and FLIM acquisitions. Moreover, the emission from these particles was stable, with no quenching observed. Thus, their photochemistry was reliable for conducting such advanced microscopy.

Insulin does not dissolve in water and requires acidic conditions for solubilizing. Thus, insulin solutions at pH 3, comparable to gastric juice pH, which inadvertently act as a barrier to the development of oral insulin formulations, were chosen for this study. It is worth noting that the isoelectric point of human insulin is 5.2.^21^ Thus, the (net) charge of an insulin molecule at pH 3, as in these samples, was cationic. It might explain the higher variation in fluorescence lifetimes observed in insulin agglomerates when in contact with amine-terminated (cationic) particles. An increased acidity, with a pH of 3, allowed more protonation of the particulate amine groups. It is known that the fluorescence lifetime of a fluorophore like FITC depends on multiple factors, including solvent polarity,^22^ ionic strength,^23^ pH,^24^ presence of quenching species in the vicinity,^25^ and molecular conformation.^26^ These factors cumulatively influence both the radiative and non-radiative decay processes, and, in turn, affect the fluorescence lifetime.

Our data suggested that the interactions between insulin and the polystyrene particles were surface-dependent, as indicated by fluctuations in the fluorescence lifetime in insulin agglomerates containing trapped amine-terminated particles, but not in those containing acid-terminated particles. The protonated amine groups interacted with the π-electron-rich aromatic amino acids in insulin, such as phenylalanine, tyrosine, and tryptophan.^27–29^ Furthermore, electrostatic and hydrophobic interactions, and hydrogen bonding might have also contributed to an extended fluorescence lifetime.^30^ Such heterogeneous interactions between the insulin and amine-terminated particles explained the disparity of fluorescence lifetimes noticed within the agglomerates. In contrast, the interactions between insulin and acid-terminated particles were subtler, with fluorescence lifetime fluctuations less pronounced.

The polymeric surface, upon interaction with insulin, triggered agglomeration and altered its photochemistry, a relevant finding from a therapeutic perspective. Previously published results from our lab, based on transmission electron microscopy, indicated insulin fibrillation in agglomerates.^5^ Our data show that, under acidic conditions, amine-terminated particles altered the biochemistry of insulin agglomerates, causing a notable fluctuation in the local pH (reflected in FITC fluorescence lifetimes) that extended beyond the surface-adsorbed insulin layer into the agglomerate matrix. Such pH fluctuation in its biochemical microenvironment indicated (potential) molecular alterations in insulin, including changes in conformation and electronic states. The insulin layer adsorbed on the particles showed higher lifetimes (amine-terminated particles > acid-terminated particles) than insulin not in contact with these particles. Interestingly, both the amine- and acid-terminated particles recorded a positive ZP at pH 3. For the amine-terminated particles, changing the solvent pH from 7 (water) to 3 also increased the (positive) ZP due to an increased protonation of the amine groups. Similarly, the acid-terminated particles, which exhibited a negative ZP in water, also showed a positive shift in ZP at pH 3, leading to a (mild) charge reversal (anionic to cationic).

This may be due to an abundance of protons (H^+^) at pH 3 that protonated the polar acid (–COO^−^) groups—thus masking the negative charge—while some of the protons were adsorbed on the particles. Hence, in this study conducted at pH 3, insulin interacted primarily with two cationic particles rather than with one cationic (amine-terminated) and one anionic (acid-terminated), as was the case in water. However, the surface chemistry of these particles differed due to the presence of disparate surface groups (amine and acid), likely resulting from differences in surface charge density and charge distribution, leading to noticeable biochemical changes in insulin or in the ZP results. Our results confirmed that the insulin indeed interacted with polystyrene surfaces, an important finding due to the widespread use of polystyrene or similar polymeric substrates in therapeutics, while the nature and impact of these interactions were determined by a complex interplay of multiple factors, including pH, surface charge, and the chemistry of surface groups.

Finally, whether such biochemical changes in insulin, resulting from exposure to functionalized surfaces, impact its bioactivity remains open to debate. However, any molecular-level changes in a therapeutically relevant peptide like insulin, especially those involving molecular conformation, which seems to be the case here, are a definite point of concern. Insulin, deserves even closer inspection, given the importance of exercising firm control over its bioactivity and bioavailability when treating diabetes patients. An uncontrolled, unmapped, and unpredictable agglomeration of insulin, with alterations to its molecular properties, carries an undeniable risk of bioactivity attenuation with additional side effects. Unfortunately, it is not easy to conduct *in vitro* bioactivity assays or *in vivo* studies on surface-adsorbed insulin (or any peptide *per se*), especially in agglomerated states, because separating/de-adsorbing these strongly adhered layers from the surfaces would require further chemical processing, which may induce molecular changes in insulin. Such protocols would also require additional purification and dose standardization steps, which can be cumbersome and, from a therapeutic perspective, invalid. It is for similar reasons that plans to investigate the fibrillation kinetics in these agglomerates, for example, using the Thioflavin-T assay, were dropped. The research community should seek refined and optimized platforms to investigate the bioactivity of insulin under similar exposure conditions, the physicochemical complexity and (potential) therapeutic impact of which are undeniably the two most relevant take-home messages from this study.

## 5. Conclusion

A biophotonic investigation based on CLSM and FLIM was undertaken to study how fluorescent amine- and acid-terminated polystyrene particles of 1 µm diameter interacted with FITC-labeled human insulin under acidic conditions (pH 3) comparable to human gastric juice. Both particles caused agglomeration of the FITC-insulin in a time-dependent manner, although such agglomeration was more prevalent for the amine-terminated ones. Besides, the amine-terminated particles caused considerable fluctuations in the fluorescence lifetime within FITC-insulin agglomerates, with these variations spanning the bulk of the agglomerates. On the contrary, the acid-terminated particles induced subtle alterations of FITC's fluorescence lifetime. However, FITC-insulin layers adhered to both particles exhibited higher fluorescence lifetimes, which may be due to the positive surface charge on both particles, irrespective of their functionalization, at pH 3. Such lifetime fluctuations, especially evident in amine-terminated particles, indicated biochemical changes in the FITC-insulin molecules and could exert a detrimental impact on insulin's bioactivity, including its ability to lower blood sugar in diabetes patients. Given the widespread use of synthetic polymers like polystyrene in the healthcare sector and pharmacy labs, such a predicament due to surface exposure cannot be ruled out and deserves further investigation.

## Conflicts of interest

The authors declare no conflict of interest.

## Supplementary Material

RA-016-D5RA08501B-s001

RA-016-D5RA08501B-s002

RA-016-D5RA08501B-s003

## Data Availability

Data available upon reasonable request from the authors. Supplementary information (SI) is available. See DOI: https://doi.org/10.1039/d5ra08501b.
