# Supplementary material for: To contact or not: an investigation into the polymeric surface interactions with human insulin and their therapeutic implications
Source: RSC Adv. 2026 Feb 12;16(10):8584–92. doi: 10.1039/d5ra08501b (PMC12895241; doi:10.1039/d5ra08501b)
Supplement: RA-016-D5RA08501B-s003 [file RA-016-D5RA08501B-s003.pdf]

Supplementary Information (SI) for RSC Advances.
This journal is © The Royal Society of Chemistry 2026

Supplementary Information

To Contact or Not: An Investigation into the Polymeric Surface Interactions with Human Insulin and their Therapeutic Implications

Megren H. A. Fagihi,[1,2,3] Chanaka Premathilaka,[4] Laura Zopf,[2,5] Tiina O'Neill,[2] Massimiliano Garré,[6] and Sourav Bhattacharjee[2,5,7,8,9,*]

[1] School of Medicine, University College Dublin, Belfield, Dublin 4, Ireland

[2] Conway Institute of Biomolecular and Biomedical Research, University College Dublin, Belfield, Dublin 4, Ireland

[3] Clinical Laboratory Sciences Department, College of Applied Medical Sciences, Najran University, Najran 55461, Kingdom of Saudi Arabia

[4] Institute of Veterinary Medicine and Animal Sciences, Estonian University of Life Sciences, Tartu 51006, Estonia

[5] School of Veterinary Medicine, University College Dublin, Belfield, Dublin 4, Ireland

[6] Super-Resolution Imaging Consortium, Royal College of Surgeons in Ireland University of Medicine and Health Sciences, Dublin D02 YN77, Ireland

[7] UCD One Health Centre, University College Dublin, Belfield, Dublin 4, Ireland

[8] UCD Earth Institute, University College Dublin, Belfield, Dublin 4, Ireland

[9] Institut für Funktionelle Anatomie, Charité – Universitätsmedizin Berlin, Campus Charité Mitte, Philippstraße 11, 10115 Berlin, Germany

*Corresponding author

Email: sourav.bhattacharjee@ucd.ie

T.: +353 1 716 6271

Supplementary Information S1. A 3D rendering of a dynamic scene showing the interactions between a FITC-insulin agglomerate (0.25 mg/mL) and amine-terminated particles (t = 48 h). A 10 μm scale bar is shown.

Supplementary Information S2. A 3D rendering of large insulin agglomerates (0.25 mg/mL) with entrapped acid-terminated polystyrene particles (t = 48 h).
